# Assessing Nutrient Intake and Nutrient Status of HIV Seropositive Patients Attending Clinic at Chulaimbo Sub-District Hospital, Kenya

**DOI:** 10.1155/2012/306530

**Published:** 2012-09-11

**Authors:** Agatha Christine Onyango, Mary Khakoni Walingo, Grace Mbagaya, Rose Kakai

**Affiliations:** ^1^Department of Nutrition and Health, School of Public Health and Community Development, Maseno University, Private Bag-40100 Maseno, Kenya; ^2^Department of Family and Consumer Sciences, School of Agriculture and Biotechnology, Moi University, P.O. Box 1125-30100 Eldoret, Kenya; ^3^Department of Biomedical Sciences and Technology, School of Public Health and Community Development, Maseno University, Private Bag-40100 Maseno, Kenya

## Abstract

*Background*. Nutritional status is an important determinant of HIV outcomes. *Objective*. To assess the nutrient intake and nutrient status of HIV seropositive patients attending an AIDS outpatient clinic, to improve the nutritional management of HIV-infected patients. *Design*. Prospective cohort study. *Setting*. Comprehensive care clinic in Chulaimbo Sub-District Hospital, Kenya. *Subjects*. 497 HIV sero-positive adults attending the clinic. *Main Outcome Measures*. Evaluation of nutrient intake using 24-hour recall, food frequency checklist, and nutrient status using biochemical assessment indicators (haemoglobin, creatinine, serum glutamate pyruvate (SGPT) and mean corpuscular volume (MCV)). *Results*. Among the 497 patients recruited (M : F sex ratio: 1.4, mean age: 39 years ± 10.5 y), Generally there was inadequate nutrient intake reported among the HIV patients, except iron (10.49 ± 3.49 mg). All the biochemical assessment indicators were within normal range except for haemoglobin 11.2 g/dL (11.4 ± 2.60 male and 11.2 ± 4.25 female). *Conclusions*. Given its high frequency, malnutrition should be prevented, detected, monitored, and treated from the early stages of HIV infection among patients attending AIDS clinics in order to improve survival and quality of life.

## 1. Introduction

There exists a complex interaction between human immunodeficiency virus (HIV), infection and immune function, with a dominant effect of HIV infection on nutritional status [1]. The influence of nutrition on immune function generally shows that suboptimal nutrition results in immunological deficiencies [1]. Nutrient deficiencies cause immunosuppression and increase susceptibility to infections with a resultant loss of immune cell function which allows intrusion by several different infectious agents. The result is reduction of the ability of the body to fight disease and subsequent acquisition of opportunistic infections. 

Nutrients that play a big role in immune function include protein, total energy, lipids, amino acids, vitamins, and minerals [2]. Optional nutrition alters immunological function and therefore disease states. This interaction affects the body's immune status predisposing one to infections. Proteins play roles as structural components of tissues and also antibodies, cytokines, acute-phase proteins, components of the complement pathways, transcription factors and enzymes. Deficiency of protein could lead to immunologically important changes in enzyme-dependent activation, antioxidant protection, complement activation, antibody-mediated virus neutralization and intercellular communication via cytokines [2]. During HIV infection, there is faster metabolism and increased energy expenditure which results in weight loss that tends to be in the form of lean tissue, such as muscle. Weight loss strongly predicts illness or death among people with HIV [3].

When macronutrient intake is insufficient to meet metabolic needs, protein-calorie malnutrition (PCM) and deficiencies of micronutrients develop [4]. These deficiencies impair both the synthesis of molecules necessary for the immune response and the function of immune-related enzyme systems [3]. In HIV disease, the presence of malnutrition strongly predicts patient survival independent of CD4 (cluster of differentiation) T-lymphocyte counts [3]. Clinical deficiencies of some nutrients occur rapidly in response to dietary deficiencies, malabsorption, or altered metabolism, while those having a storage form in the body may take longer. The presenting symptoms of malnutrition typically include weight loss, a change in body habitus (loss of lean body mass) or a change in functional status (inability to perform daily activities) [4].

## 2. Materials and Methods 

This prospective study was performed in the Comprehensive Care Clinic of the Chulaimbo Sub-District Hospital, Kisumu West District, Kenya. The study population was selected among HIV-seropositive patients attending the outpatient clinic from February 10th to July 16th, 2010, with their informed consent. The study was performed two days a week, the days being selected from a table of random numbers. Patients requiring hospitalization were excluded. Each patient was recruited once only, on his or her next visit and followed for six months. The investigation was performed with the help of research assistants. Demographic data and information on risk factors for HIV infection and malnutrition were collected using a standardized questionnaire. Diet variety was assessed using a food frequency checklist once at the start of the study and nutrient intake using 24-hour dietary recall every month for six month. Creatinine, haemoglobin, Serum glutamate pyruvate transaminase (SGPT) and mean corpuscular volume (MCV) measures were used to assess the nutrient status of the patients at the start of the study and at the end of the study. Ethical clearance was obtained from Institutional Ethics Research Committee, Moi University Teaching Hospital (FAN: IREC 000470). The analysis of the nutrient value of the foods consumed within 24 hours was done by the use of the food composition tables by Sehmi [5]. A value of over 100% was considered as above RDA nutrient consumption, whereas those consuming 90%–100% was considered optimum nutrient consumption and those consuming <90% considered as below RDA nutrient consumption. Statistical analysis was performed using the Chi-square test, *t*-test, and analysis of variance. Analyses were conducted using SPSS version 16. Differences were considered significant if *P* < 0.05.

## 3. Results 

On average there were three meals served per day. Majority of the patients had an average of three meals per day in the month of February 63 (22.0%) males and 212 (77.1%) females. The frequency reduced in the month of July 52 (23.3%) males and 172 (76.8%) females as illustrated in Table 1. An examination of the mineral and vitamin intake as shown in Table 2 revealed that the mean intake was below the RDA, except for iron in the males (10.35 ± 3.49) and thiamine in both male and female (1.63 ± 0.66 male, and 1.76 ± 0.69 female). 

The foods consumed among the HIV seropositive patients contained the following nutrients energy, proteins, calcium, iron, vitamin A, vitamin C, thiamine, riboflavin, and niacin as shown in Table 3. Foods were classified into the following sources: cereals, animal and animal products, legumes, vegetable, fruit, fats/oils, and beverages. Majority had variety of vegetables 1442 (23.8%) and fat/oil 493 (49.6%). There was variety of foods consumed as shown in Table 4.

Haemoglobin, creatinine, SGPT, and MCV indicators were assessed. Mean haemoglobin was 11.2 g/dL (11.4 ± 2.60 male and 11.2 ± 4.25 female), creatinine 0.63 mg/dL (0.728 ± 0.22 males and 0.604 ± 19.14 females), SGPT 24.8 IU/L (27.7 ± 20.21 males and 24.1 ± 18.78 females), and MCV 85.0 femtoliters (86.6 ± 15.93 males and 84.6 ±14.51 females) as shown in Table 5. 

There was a significant difference in the mean of creatinine between the male and female patients (*P* < 0.0001). The male had higher creatinine level compared to the female (0.73 ± 0.22 male and 0.60 ± 19.14 female). Though the female had a higher intake of the most nutrients, there was no statistical significant difference in the mean nutrient intake except in thiamine (*P* = 0.024), as shown in Table 6. 

## 4. Discussion 

Nutritional quality of the diet does improve with consumption of greater food diversity, [6, 7]. Diet diversity has however widely been associated with high socioeconomic status [8]. This is because people with high income may have the economic ability to purchase different types of foods from different food groups whereas those with low income stick to the few cheaper foods available, and this limits diet diversification among the poor people [9]. Monthly income can be a strong and significant predictor of diet diversity among HIV patients. This is true in this study as the monthly income was below the average of one dollar (1USD = 80) a day, [10] and majority had an inadequate dietary intake. Studies have shown that diet diversity correlates with nutrient adequacy. Hatloy et al. [7] and Slattery et al. [8] observed that nutritional quality of the diet does improve with consumption of greater food diversity. A study by Stewart [11] reported that daily servings of the same food from each food source may not be enough, but that one should choose variety within food sources because the characteristic nutrients in each group vary greatly for individual foods. 

The diet of HIV positive patients even if it is diversified is not sufficient to reach the recommended RDA in the sample analyzed in this study. Findings of this study showed that there was variety in the intake of foods from various food sources even though the amounts were not sufficient among the HIV seropositive patients, Table 4. This supports the findings of Friis [12] in which a diversified diet contributed to resistance to opportunistic infections in AIDS patients. However it is generally understood that no food contains all necessary nutrients and that diversity in the diet is needed to ensure a balanced diet. This implies that diversified diets are likely to ensure nutrient adequacy, and individuals who diversify diets have a likelihood of having a good nutritional status. The most commonly consumed food sources were vegetables (23.8%) and fats or oils (49.6%). The foods consumed and the frequency of consumption determines an individual's food security status [13]. Therefore if the food consumption and frequency are low the HIV seropositive patient becomes more food insecure. The results suggests that majority of the patients consumed a variety of foods from the vegetables sources daily and this may be due to the vegetables being available and affordable compared to the other sources of food like animal proteins. 

There was inadequate nutrient intake reported in most of the patients although majority (55.3%) had three meals per day. In the month of June majority of the patients had two meals (42.9%), Table 1. Adequate diet is vital for the health and survival of all HIV-infected persons and this reduces immunosuppression. As observed in this study, the mean energy intake is lower than the RDA for both male and female patients as shown in Table 2. The patients may have been taking variety of carbohydrates sources but not in adequate amounts to meet their dietary needs. This does not agree with findings as reported by Hogg et al. [14] that in a study in South Africa most HIV-infected patients had energy and protein intakes that met at least 67% of the recommended daily amount. However, most of these patients had indications of low intake of vitamins C, A, D, and B_6_ and of zinc, iron, and calcium. This is probably due to their high consumption of carbohydrate sources like maize meal, which contains only low levels of these nutrients. The results in this study are consistent with findings by Onyango et al. [15] that there was a lower intake of energy as well as most of the other nutrients among HIV seropositive patients. Energy intake is related to the stage of the infection, rapid weight loss, anorexia, opportunistic infections, malabsorption, and altered metabolism [16]. Thus it is clear that HIV-infected patients are at increased nutritional risk. Energy requirements increase significantly as the HIV disease progresses [17]. This may thus be viewed as a bad trend for patients with low energy intake as reported in this study. The higher energy intake assists to a certain degree in reducing wasting and improves the well-being of the patients [1].

Both male and female patients reported a mean protein intake of 41.65 g (±15.67) and 39.23 g (±11.89) lower than the RDA of 63 g and 50 g as illustrated in Table 2. The results differ from the findings by Watson [18], which reports a mean protein intake in HIV-positive patients was higher than the RDA. Studies carried out on HIV-infected patients in the Free State province of South Africa and in Boston (USA) reported that majority of the patients had a protein intake that met at least 67% of the RDA [19]. The low intake of protein observed in this study may be associated with the patient's economic ability. It was envisaged that the low protein intake reported in this study may not compensate for the increased urinary nitrogen loss, increased protein utility, decreased skeletal protein synthesis, and increased skeletal muscle breakdown that is reported in HIV-infected individuals. 

In this study, the majority of patients had an inadequate or low intake of micronutrients that was <90% or lower than the RDA. The study also established that the intake of calcium and iron (females) was <90% of the RDA and vitamins, such as A, C, riboflavin and niacin in the male patients. The female patients had higher intake of most of the nutrients compared to the males except iron as illustrated in Table 3. Studies have shown that some minerals/trace elements may be key factors in maintaining health despite human immunodeficiency virus infection and in reducing mortality. Values higher than the RDA were identified for thiamine (male 135.8% and female 160%) and iron in male (128.8%) in this study population. Each of the mineral/trace elements examined in this study may contribute to the general well-being of HIV-infected persons. Calcium has been shown to reduce diarrhoea in HIV-positive/AIDS patients [20]. In this study, the mean calcium intake in male patients 487.57 ± 306.15 and in female patients 542.21 ± 320.59 was lower than the RDA (1200 mg). Therefore, the results obtained from this study tend to suggest that patients with a high dietary intake might be able to replace lost calcium and in turn reduce the burden of diarrhoea. Iron is essential for the formation and functioning of red blood cells, and vitamin C is known to promote the absorption of iron. In male patients, the iron intake was higher (10.49 ± 3.49) than the RDA, nonetheless the female had a lower intake as shown in Table 2. Iron is one of the micronutrients that is commonly deficient in HIV infection. Haemoglobin level (11.405 mg/dL (±2.60) in male and 11.185 mg/dL (±4.25) in female) was determined in this study so as to verify its correlation with dietary intake. As mentioned earlier, the female population had an inadequate intake of iron as opposed to the male. The reason for this discrepancy is not clear, but it may be related to the fact that iron is lacking in women's food due to a lack of knowledge, consuming foods/diets deficient in iron or because of extra demands during the menstrual cycle (women need extra iron until they pass the menopause stage). 

Tang et al. [21] observed a slower progression of disease and reduced risk of mortality with an increased intake of riboflavin, thiamine, and vitamin C. Vitamin C has been found to affect immune function in several ways [17]. It can stimulate the production of interferons; one of the protein that protects cells against viral attack. However, the dietary intake of vitamin C was inadequate among the patients in this study. There is evidence that increased intakes of vitamin C may help to reduce the risk of diseases associated with increased oxidative stress [17]. It is therefore envisaged that the inadequate vitamin C intake reported among the patients in this study is not beneficial to the patients. Although, the greater percentage of patients in this study had a dietary intake of the thiamine higher than the RDA, it should be noted that even a mild state of deficiency of these vitamins could result in an altered immune function, especially in patients who are not on antiretroviral drugs. As HIV infection progresses, coupled with opportunistic infections and metabolic demand, HIV-infected individuals may be unable to meet their required nutritional needs. This may be due to decreased oral intake, decreased nutrient absorption, increased nutrient requirements and changes in metabolism and nutrient transport, which could steadily result in greater inadequacy of these vitamins. The dietary intake of vitamin A in this study was lower than the RDA for a greater percentage of the patients (22.8% male and 26.7% female). Tang et al. [21] reported that 12%–19% of HIV-positive patients at various stages of HIV infection show vitamin A inadequacy that is more prevalent in women than in men. However this study established that the females had a higher intake of vitamin A compared to the males. Studies have shown that there is a relationship between the dietary intake of vitamin A and immune function [22]. High dietary intake of vitamin A may be related to metabolic demand during the acute phase of HIV infection, or an increased dietary intake, while the low intake could probably be associated with a more rapid progression to AIDS [1]. There were low levels of haemoglobin (males 11.41 ± 2.60 and females 11.19 ± 4.25) and creatinine, SGPT and MCV levels were at the cut off for majority of the female patients, Table 5. However, this study was performed in a hospital setting and therefore results may not be generalizable and may present a selection bias.

## 5. Conclusion 

Inadequate dietary intake could contribute to the severity of HIV-1 infection and to the depletion of CD_4_^+^ T-cell population in addition to the oxidative stress that might arise from depletion of antioxidant molecules. It is therefore recommended that adequate dietary intake and nutrient supplementation should be encouraged in HIV infection. Given its high frequency among patients attending AIDS clinics, malnutrition should be prevented, detected, monitored, and treated from the early stages of HIV infection, using standardized procedures, as these simple measures may improve both survival and quality of life. 

## Figures and Tables

**Table 1:** Distribution of HIV seropositive patients by frequency of meals and sex.

Month	Sex	None	One	Two	Three
		No. (%)	No. (%)	No. (%)	No. (%)
*n* = 497					
February	Male	0 (0%)	9 (19.6%)	33 (19.0%)	63 (22.9%)
	Female	2 (100%)	37 (80.4%)	141 (81.0%)	212 (77.1%)

*n* = 495					
March	Male	1 (50%)	3 (11.5%)	43 (19.8%)	58 (23.2%)
	Female	1 (50%)	23 (88.5%)	174 (80.2%)	192 (76.8%)

*n* = 493					
April	Male	0 (0%)	9 (23.1%)	47 (21.8%)	49 (20.6%)
	Female	0 (0%)	30 (76.9%)	168 (78.2%)	190 (79.4%)

May	Male	0 (0%)	9 (23.1%)	42 (18.5%)	63 (22.9%)
	Female	0 (0%)	30 (76.9%)	185 (81.5%)	164 (77.1%)

June	Male	0 (0%)	4 (11.4%)	52 (21.2%)	49 (23.0%)
	Female	0 (0%)	31 (88.6%)	193 (78.8%)	164 (77.0%)

July	Male	0 (0%)	5 (16.7%)	48 (20.1%)	52 (23.2%)
	Female	0 (0%)	25 (83.3%)	191 (79.9%)	172 (76.8%)

**Table 2:** HIV seropositive patients by mean nutrient intake and sex.

Nutrients	Sex and RDA	Mean SD (±)	February n = 497	March n = 495	April n = 493	May n = 493	June n = 493	July *n* = 493	Average
Protein (g)	Male 56	Mean SD	40.43 ± 11.41	41.51 ± 15.19	42.53 ± 15.42	42.54 ± 14.93	40.16 ± 12.48	42.73 ± 14.76	41.65 ± 15.67
	Female 46	Mean SD	40.83 ± 11.23	39.02 ± 11.90	39.93 ± 11.86	38.89 ± 10.97	37.88 ± 9.96	38.81 ± 10.93	39.23 ± 11.89

Iron (mg)	Male 8	Mean SD	10.35 ± 3.40	10.48 ± 3.17	10.48 ± 3.45	10.52 ± 3.06	10.57 ± 2.97	10.56 ± 3.01	10.49^a^ ± 3.49
	Female 18	Mean SD	10.54 ± 3.29	10.46 ± 3.39	10.43 ± 3.28	10.41 ± 3.24	10.50 ± 3.10	10.44 ± 3.21	10.47 ± 3.40

Calcium (mg)	Male 1200	Mean SD	415.10 ± 280.77	500.20 ± 312.50	499.98 ± 323.64	499.86 ± 324.6	507.59 ± 312.00	502.71 ± 321.33	487.57 ± 306.15
	Female 1200	Mean SD	561.41 ± 320.30	541.40 ± 319.47	538.58 ± 324.31	534.66 ± 321.96	542.89 ± 310.48	534.33 ± 317.74	542.21 ± 320.59

Vit A (IU)	Male 17000	Mean SD	3874.14 ± 4468.34	4039.83 ± 4390.48	3999.07 ± 4422.36	4245.36 ± 4370.88	4281.56 ± 4340.79	4265.56 ± 4353.56	4117.59 ± 4347.50
	Female 17000	Mean SD	4535.39 ± 6510.64	5150.39 ± 6766.37	4935.38 ± 6741.48	5119.59 ± 6662.64	5157.73 ± 6636.02	5138.88 ± 6649.87	5006.23 ± 6660.74

Vit C (mg)	Male 90	Mean SD	49.54± 28.38	52.05 ± 27.22	51.78 ± 26.84	50.38 ± 27.69	49.97 ± 24.48	51.01 ± 27.25	52.01 ± 28.28
	Female 75	Mean SD	47.06 ± 26.84	54.66 ± 23.64	52.79 ± 25.05	53.08 ± 25.21	52.70 ± 24.06	52.92 ± 24.82	52.20 ± 24.93

Thiamine (mg)	Male 1.2	Mean SD	1.63 ± 0.52	1.68 ± 0.73	1.68 ± 0.72	1.66 ± 0.68	1.58 ± 0.62	1.68 ± 0.67	1.65^a^ ± 0.66
	Female 1.1	Mean SD	1.76 ± 0.66	1.73 ± 0.71	1.78 ± 0.81	1.72 ± 0.68	1.60 ± 0.61	1.73 ± 0.67	1.72^a^ ± 0.69

Riboflavin (mg)	Male 1.3	Mean SD	0.44 ± 0.35	0.44 ± 0.41	0.42 ± 0.74	0.46 ± 0.42	0.46 ± 0.42	0.47 ± 0.42	0.45 ± 0.43
	Female 1.1	Mean SD	0.42 ± 0.38	0.42 ± 0.39	0.43 ± 0.42	0.47 ± 0.43	0.46 ± 0.43	0.48 ± 0.43	0.45 ± 0.43

Niacin (mg)	Male 16	Mean SD	10.21 ± 3.40	10.49 ± 4.62	10.63 ± 4.46	10.08 ± 3.55	10.08 ± 3.56	10.05 ± 3.62	10.38 ± 3.96
	Female 14	Mean SD	10.86 ± 4.47	10.63 ± 4.69	10.75 ± 4.56	10.15 ± 3.99	10.16 ± 3.97	10.12 ± 4.03	10.45 ± 4.52

^a^ Above RDA.

**Table 3:** Determination of adequacy of nutrient intake among HIV seropositive patients (*n* = 497).

Nutrients	Sex	No. (%)
Proteins (grams)	Male	72.1%
	Female	88.7%

Calcium (milligrams)	Male	34.6%
	Female	38.5%

Iron (milligrams)	Male	128.8%^a^
	Female	58.3%

Vitamin A (IU)	Male	22.8%
	Female	26.7%

Vitamin C (milligrams)	Male	55.0%
	Female	62.7%

Thiamine (milligrams)	Male	135.8%^a^
	Female	160.0%^a^

Riboflavin (milligrams)	Male	33.8%
	Female	38.4%

Niacin (milligrams)	Male	63.8%
	Female	77.6%

^a^ Above RDA.

This proportion is obtained by dividing the total nutrient intake by the RDA multiplied by 100 percent. Nutrient intake/RDA × 100%.

**Table 4:** Food variety of HIV seropositive patients (*n* = 497).

Sources	Daily	Weekly	Occasionally	Never
	No. (%)	No. (%)	No. (%)	No. (%)
Cereal	769 (15.5%)	681 (13.7%)	2707 (54.4%)	813 (16.4%)
Animal and animal products	286 (7.3%)	458 (11.5%)	2201 (55.4%)	1026 (25.8%)
Legume	250 (10.1%)	346 (13.9%)	1421 (57.2%)	468 (18.8%)
Vegetables	1442 (23.8%)	966 (15.9%)	2690 (44.5%)	963 (15.8%)
Fruits	358 (10.3%)	411 (11.8%)	2239 (64.4%)	471 (13.5%)
Fats/oil	493 (49.6%)	50 (5.0%)	200 (20.1%)	251 (25.3%)
Beverage	587 (23.6%)	159 (6.4%)	1231 (49.5%)	508 (20.5%)

**Table 5:** Distribution of HIV sero-positive patients by biochemical indicators and sex.

Biochemical assessment indicators	Sex	*N*	Mean ± (SD)	Normal range
Haemoglobin (g/dL)	Male	105	11.41 (±2.60)	14–18
	Female	392	11.19 (±4.25)	12–16

Creatinine (mg/dL)	Male	105	0.73 (±0.22)	0.6–1.5
	Female	392	0.60 (±19.14)	0.6–1.5

SGPT (IU)	Male	105	27.71 (±20.21)	0–50
	Female	392	24.06 (±18.78)	0–50

MCV (femtoliters)	Male	105	86.63 (±15.93)	79–100
	Female	392	84.61 (±14.51)	79–100

Key: SGPT: serum glutamic pyruvate transaminase, MCV: mean corpuscular volume.

**Table 6:** The difference in mean values of nutrient intake and nutrient status between males and females (*n* = 497).

Nutrient status and nutrient intake indicators	*t* for equivalence of means	df	Sig
Haemoglobin (g/dL)	0.661	269.232	0.509
Creatinine (mg/dL)	5.3444	150.865	0.000*
SGPT (UI/L)	1.666	155.460	0.098
MCV (femtoliters)	1.179	153.349	0.240
Protein (g)	−0.323	160.926	0.747
Iron (mg)	−0.526	159.699	0.599
Calcium (mg)	−1.455	183.227	0.147
Vitamin A (IU)	−1.211	235.640	0.227
Vitamin C (mg)	0.806	157.390	0.421
Thiamine (mg)	−2.267	205.178	0.024*
Riboflavin (mg)	0.451	175.100	0.652
Niacin (mg)	−1.615	210.635	0.108

Key: *α = 0.05, SGPT: serum glutamic pyruvate transaminase, MCV: mean corpuscular volume.
